# The rs429358 Locus in Apolipoprotein E Is Associated With Hepatocellular Carcinoma in Patients With Cirrhosis

**DOI:** 10.1002/hep4.1886

**Published:** 2021-12-27

**Authors:** Hamish Innes, Hans Dieter Nischalke, Indra Neil Guha, Karl Heinz Weiss, Will Irving, Daniel Gotthardt, Eleanor Barnes, Janett Fischer, M. Azim Ansari, Jonas Rosendahl, Shang‐Kuan Lin, Astrid Marot, Vincent Pedergnana, Markus Casper, Jennifer Benselin, Frank Lammert, John McLauchlan, Philip L. Lutz, Victoria Hamill, Sebastian Mueller, Joanne R. Morling, Georg Semmler, Florian Eyer, Johann von Felden, Alexander Link, Arndt Vogel, Jens U. Marquardt, Stefan Sulk, Jonel Trebicka, Luca Valenti, Christian Datz, Thomas Reiberger, Clemens Schafmayer, Thomas Berg, Pierre Deltenre, Jochen Hampe, Felix Stickel, Stephan Buch

**Affiliations:** ^1^ School of Health and Life Sciences Glasgow Caledonian University Glasgow United Kingdom; ^2^ Population and Lifespan Sciences School of Medicine University of Nottingham Nottingham United Kingdom; ^3^ Public Health Scotland Glasgow United Kingdom; ^4^ Department of Internal Medicine I University Hospital University of Bonn Bonn Germany; ^5^ National Institute for Health Research (NIHR), Nottingham Biomedical Research Centre Nottingham University Hospitals National Health Service Trust and the University of Nottingham Nottingham United Kingdom; ^6^ Department of Gastroenterology and Hepatology University Hospital Heidelberg Heidelberg Germany; ^7^ Department of Internal Medicine IV Medical University of Heidelberg Heidelberg Germany; ^8^ Peter Medawar Building for Pathogen Research Nuffield Department of Medicine and the Oxford NIHR Biomedical Research Centre Oxford University Oxford United Kingdom; ^9^ Division of Hepatology Department of Medicine II Laboratory for Clinical and Experimental Hepatology Leipzig University Medical Center Leipzig Germany; ^10^ Medical Department 1 University Hospital Halle Martin‐Luther Universität Halle‐Wittenberg Halle Germany; ^11^ Division of Gastroenterology and Hepatology Centre Hospitalier Universitaire Vaudois Université de Lausanne Lausanne Switzerland; ^12^ Department of Gastroenterology and Hepatology Centre Hospitalier Universitaire UCLouvain Namur Université Catholique de Louvain Yvoir Belgium; ^13^ Laboratoire MIVEGEC Montpellier France; ^14^ Department of Medicine II Saarland University Medical Center Saarland University Homburg Germany; ^15^ Medical Research Council‐University of Glasgow Centre for Virus Research Glasgow United Kingdom; ^16^ Center for Alcohol Research University of Heidelberg Heidelberg Germany; ^17^ Medical Department Salem Medical Center Heidelberg Germany; ^18^ Department of Internal Medicine III Division of Gastroenterology and Hepatology Medical University of Vienna Vienna Austria; ^19^ Department of Internal Medicine, General Hospital Oberndorf, Teaching Hospital of the Paracelsus Medical University Salzburg Oberndorf Austria; ^20^ Department of Clinical Toxicology Klinikum Rechts der Isar Technical University of Munich Munich Germany; ^21^ Department of Medicine University Medical Center Hamburg‐Eppendorf Hamburg Germany; ^22^ Department of Gastroenterology, Hepatology, and Infectious Diseases Otto‐von‐Guericke University Hospital Magdeburg Germany; ^23^ Department of Gastroenterology, Hepatology, and Endocrinology Hannover Medical School Hannover Germany; ^24^ Department of Medicine I University Hospital Schleswig Holstein–Campus Lübeck Lübeck Germany; ^25^ Medical Department 1 University Hospital Dresden Technische Universität Dresden Dresden Germany; ^26^ Department of Internal Medicine I Goethe University Frankfurt Germany; ^27^ European Foundation for Study of Chronic Liver Failure Barcelona Spain; ^28^ Precision Medicine–Department of Transfusion Medicine and Hematology Fondazione IRCCS Ca’ Granda Ospedale Maggiore Policlinico Milan Italy; ^29^ Department of Pathophysiology and Transplantation Università degli Studi di Milano Milan Italy; ^30^ Department of General, Visceral, Vascular, and Transplant Surgery Rostock University Medical Center Rostock Germany; ^31^ Department of Gastroenterology Hepatopancreatology, and Digestive Oncology University Clinics of Brussels Hospital Erasme Brussels Belgium; ^32^ Department of Gastroenterology and Hepatology Clinique St Luc Bouge Belgium; ^33^ Center for Regenerative Therapies Dresden Technische Universität Dresden Dresden Germany; ^34^ Department of Gastroenterology and Hepatology University Hospital of Zurich Zurich Switzerland

## Abstract

The host genetic background for hepatocellular carcinoma (HCC) is incompletely understood. We aimed to determine if four germline genetic polymorphisms, rs429358 in apolipoprotein E (*APOE*), rs2642438 in mitochondrial amidoxime reducing component 1 (*MARC1*), rs2792751 in glycerol‐3‐phosphate acyltransferase (*GPAM*), and rs187429064 in transmembrane 6 superfamily member 2 (*TM6SF2*), previously associated with progressive alcohol‐related and nonalcoholic fatty liver disease, are also associated with HCC. Four HCC case‐control data sets were constructed, including two mixed etiology data sets (UK Biobank and FinnGen); one hepatitis C virus (HCV) cohort (STOP‐HCV), and one alcohol‐related HCC cohort (Dresden HCC). The frequency of each variant was compared between HCC cases and cirrhosis controls (i.e., patients with cirrhosis without HCC). Population controls were also considered. Odds ratios (ORs) associations were calculated using logistic regression, adjusting for age, sex, and principal components of genetic ancestry. Fixed‐effect meta‐analysis was used to determine the pooled effect size across all data sets. Across four case‐control data sets, 2,070 HCC cases, 4,121 cirrhosis controls, and 525,779 population controls were included. The rs429358:C allele (*APOE*) was significantly less frequent in HCC cases versus cirrhosis controls (OR, 0.71; 95% confidence interval [CI], 0.61‐0.84; *P* = 2.9 × 10^−5^). Rs187429064:G (*TM6SF2*) was significantly more common in HCC cases versus cirrhosis controls and exhibited the strongest effect size (OR, 2.03; 95% CI, 1.45‐2.86; *P* = 3.1 × 10^−6^). In contrast, rs2792751:T (*GPAM*) was not associated with HCC (OR, 1.01; 95% CI, 0.90‐1.13; *P* = 0.89), whereas rs2642438:A (*MARC1*) narrowly missed statistical significance (OR, 0.91; 95% CI, 0.84‐1.00; *P* = 0.043). *Conclusion:* This study associates carriage of rs429358:C (*APOE*) with a reduced risk of HCC in patients with cirrhosis. Conversely, carriage of rs187429064:G in *TM6SF2* is associated with an increased risk of HCC in patients with cirrhosis.

## Graphical abstract

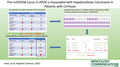

**Abbreviations**
HU: Hounsfield unit
APOA/B/E: apolipoprotein A/B/E
ArLD: alcohol‐related liver disease
AST: aspartate aminotransferase
BMI: body mass index
CI: confidence interval
CVD: cardiovascular disease
FE: finite element
GPAM: glycerol‐3‐phosphate acyltransferase
HCC: hepatocellular carcinoma
HCV: hepatitis C virus
HSD17B13: 17‐β hydroxysteroid dehydrogenase 13
ICD‐9/10: International Classification of Diseases, Ninth/Tenth Revision
LDLR: lipoprotein receptor
LOR: log odds ratio
MARC1: mitochondrial amidoxime reducing component 1
NAFLD: nonalcoholic fatty liver disease
OR: odds ratio
TM6SF2: transmembrane 6 superfamily member 2
UKB: United Kingdom Biobank

Hepatocellular carcinoma (HCC) is the third most common type of cancer death, responsible for approximately 800,000 deaths globally every year worldwide.^(^ ^1^ ^)^ Most cases of HCC develop against a background of advanced liver fibrosis and cirrhosis. Like any cancer, HCC is a product of somatic mutations acquired in pivotal driver genes but also influenced by germline (i.e., constitutional) polymorphisms modifying the susceptibility to developing HCC.^(^ ^2^ ^)^ Thus far, several such polymorphisms have been identified and robustly validated, including rs738409 in patatin‐like phospholipase domain containing 3 (*PNPLA3*), rs58542926 in transmembrane 6 superfamily member 2 (*TM6SF2*), and rs72613567 in 17‐β hydroxysteroid dehydrogenase 13 (*HSD17B13*).^(^ ^3^, ^4^, ^5^, ^6^ ^)^ However, this explains only part of the host genetic background underlying HCC development. A more complete understanding of the constitutional genetic polymorphisms that predispose patients to HCC could herald several important advancements. In the short term for example, it could support risk stratification of patients with cirrhosis with respect to HCC screening decision^(^ ^7^ ^)^; in the longer term, it could guide the discovery of chemoprevention agents if any of the risk loci prove to be “druggable.”

In a recent exome association study, Jamialahmadi et al.^(^ ^8^ ^)^ identified three novel missense variants associated with hepatic fat content. These variants were rs429358 in apolipoprotein E (*APOE*, rs2792751 in glycerol‐3‐phosphate acyltransferase (*GPAM*), and rs187429064 in *TM6SF2,* where the latter is in complete linkage equilibrium with the better known rs58542926 locus. They also show that the rs2642438 missense variant in mitochondrial amidoxime reducing component 1 (*MARC1)*, which we and others have recently identified as a risk factor for cirrhosis,^(^ ^9^, ^10^ ^)^ is associated with liver fat content, too. In a parallel study, Bianco et al.^(^ ^11^ ^)^ indicated that higher liver fat content is causally associated with HCC occurrence. On that basis, genetic factors that alter liver fat content may also alter HCC risk; these variants therefore warrant exploration in candidate gene‐association studies for HCC. To that end, our primary objective was to explore a possible association of each of these four aforementioned variants with HCC across a variety of large data sets and etiologies.

## Materials and Methods

### Scientific Approach

This study uses data from the following four HCC case‐control data sets: two mixed etiology cohorts (United Kingdom Biobank [UKB] and FinnGen), one hepatitis C virus (HCV) cohort (STOP‐HCV), and one alcohol‐related liver disease (ArLD) cohort (Dresden study).

The following four candidate variants were considered for association with HCC: 1) rs429358 (*APOE*), 2) rs2792751 (*GPAM*), 3) rs2642438 (*MARC1*), and 4) rs187429064 (*TM6SF2*). Genotyping methods for these variants are described in Supporting Materials Appendix A.

In the broadest terms, our goal was to assess if the frequency of these variants was different for HCC cases versus non‐HCC controls. Two types of non‐HCC controls were considered, patients with cirrhosis without HCC and population controls without HCC. On one hand, comparing HCC cases to cirrhosis controls is essential to eliminate confounding, i.e., because variants associated with HCC tend also to be associated with progression to cirrhosis. On the other hand, population controls lend insight because HCC can also arise in patients at a precirrhosis stage (i.e., particularly nonalcoholic fatty liver disease [NAFLD]‐related HCC).^(^ ^12^, ^13^ ^)^ A population perspective is also relevant to early detection case‐finding initiatives for HCC.^(^ ^14^ ^)^

### Case‐Control Data Sets

#### UK Biobank

The UKB is a cohort of half a million middle‐aged individuals from the United Kingdom, recruited in 2006‐2010. Blood specimens donated at enrollment have been used to characterize participants in terms of genetic factors as well as being serum biomarkers (e.g., alanine aminotransferase). Participant data are also linked to UK health registries to capture medical presentations occurring both before and after enrollment.^(^ ^15^ ^)^

This study was restricted to UKB participants of White British ancestry (UKB field ID: 22006). We then excluded those with a poor quality genetic sample (defined by UKB field ID: 22027) or who were related to another participant (inferred by a kinship coefficient ≥0.1). Cases were participants with a history of HCC, defined as a hospital admission, death, or cancer registration with HCC (International Classification of Diseases, Tenth Revision [ICD‐10]: C22.0, or ICD‐9: 155.0), either before or after UKB enrollment. Liver disease etiology for the HCC cases was estimated using a hierarchical definition of a) viral hepatitis, b) autoimmune liver disease in the absence of a, c) ArLD in the absence of a‐b, d) NAFLD in the absence of a‐c, and e) other/unknown in the absence of a‐d. Risk factors for these etiologies were discerned through a combination of hospital admissions and/or information reported during the UKB enrollment interview (Supporting Table S1).

The following two control groups were considered: ontrol group 1 included UKB participants with a hospital admission for liver cirrhosis but without a history of HCC. Hospital admissions due to cirrhosis were identified using a validated set of ICD and operation/procedure codes^(^ ^16^ ^)^ (See Supporting Table S2 for further details.) Control group 2 included all UKB participants without a history of HCC. The vast majority of individuals in this group had no history of chronic liver disease. Control group 2 was broadly equivalent to a general population control group.

#### FinnGen

FinnGen is a public–private partnership project, combining genotyping data from Finnish biobanks with electronic health record data derived from national health registries. Genome‐wide association study (GWAS) summary statistics for more than 1,800 phenotypes/endpoints, including for primary liver cancer, have been publically released.

For this study, we used the latest R4 data released (published November 2020) pertaining to a sample size of 176,899 individuals.^(^ ^17^ ^)^ Cases were individuals with a history/diagnosis of primary liver cancer (ICD‐10: C22 and ICD‐9: 155), whereas controls were all individuals without a diagnosis of primary liver cancer. Similar to the UKB control group 2, this largely comprised individuals without any preexisting liver disease. GWAS summary statistics relating specifically to HCC were not available.

### STOP‐HCV Cirrhosis Study

The STOP‐HCV cirrhosis study comprised approximately 1,200 patients with hepatitis C‐related cirrhosis. Participants were recruited from 31 specialist liver clinics in the United Kingdom between January 2015 and July 2016. Cirrhosis was defined through histologic assessment, imaging, or a validated serum biomarker consistent with liver cirrhosis (i.e., aspartate aminotransferase [AST]‐to‐platelet ratio index >2, FibroTest >0.73, or enhanced liver fibrosis score >10.48). Blood specimens collected at enrollment were used to generate host‐genotyping data through the Affymetrix UK Biobank array. Furthermore, participants from England have been linked to national hospital admission, cancer registrations, and mortality data.

The present analysis was restricted to participants from England (i.e., to ensure complete data on hospital admissions, cancer registrations, and mortality) and participants of White ethnicity. As with the UKB, cases were defined on the basis of an in‐patient hospital admission, death, or cancer registration indicating HCC (ICD‐10: C22.0; ICD‐9: 155.0) before or after study enrollment. Controls were all participants without a history of HCC.

### Dresden Alcohol HCC Cohort

The Dresden HCC cohort included 2,311 patients with a history of high‐risk alcohol consumption in whom nonalcohol‐related causes of chronic liver disease had been excluded. Patients were recruited from gastroenterology and hepatology hospitals across five European countries (Austria, France, Germany, Italy, and Switzerland). For this study, cases were patients with a diagnosis of HCC determined through histologic and/or imaging (computed tomography or magnetic resonance imaging [MRI]) investigations.

As with the UKB, two control groups were considered. Control group 1 was individuals diagnosed with alcohol‐related cirrhosis but without a history of HCC. Control group 2 comprised patients without cirrhosis.

The diagnosis of alcohol‐related cirrhosis was established as described in detail.^(^ ^18^ ^)^ Briefly, the diagnosis was based on a history of prolonged sustained alcohol intake of a minimum of 40 g/day in women and 60 g/day in men, together with histologic examination of liver tissue or compatible historical, clinical, laboratory, radiologic, and endoscopic features of advanced chronic liver disease. Patients were excluded if they had any other potential cause of liver injury, specifically if they were positive for hepatitis B surface antigen, anti‐HCV, antinuclear antibodies (titer >1:80), or antimitochondrial antibodies (titer >1:40). Patients with elevated serum ferritin concentrations and a transferrin saturation >50%, a serum ceruloplasmin concentration <20 mg/dL (0.2 g/dL), or a serum alpha‐1 antitrypsin concentration <70 mg/dL (13 µmol/L) were further investigated and excluded, as appropriate. The diagnosis of HCC was based on histologic examination of tumor tissue or evidence on imaging, preferably using two modalities, of lesions that were hypervascular in the arterial phase with washout in the portal venous or delayed phases.^(^ ^19^ ^)^

Patients with alcohol misuse but no evidence of cirrhosis (control group 2) were recruited as described in detail.^(^ ^15^ ^)^ In brief, these patients had a background of alcohol consumption of at least 60 g/day for ≥10 years with or without features of alcohol dependence^(^ ^20^ ^)^; none had historical, clinical, or laboratory evidence of cirrhosis as reflected by AST‐adapted cut‐off values for liver stiffness measured by transient elastography, as described.^(^ ^21^ ^)^

All participants from the Dresden cohort were of Caucasian ancestry, and genotyping was performed using the Illumina BeadChip array (see Supporting Materials Appendix A). The study protocol was approved by the ethics committees of the participating institutions, and all patients provided written informed consent before study inclusion.

### Data Analysis

#### Association With Liver Fat Content

We started by replicating the UKB association between each candidate variant and liver fat fraction, as reported by Jamialahmadi et al.^(^ ^8^ ^)^ This allowed us to compare each variant’s direction of association with liver fat content with the direction of association for HCC. Liver fat fraction was measured through MRI, which at the time of analysis was available for a subset of 9,893 participants (UKB Field ID: 22436). We performed log10 transformation on this variable to achieve approximate normality. Covariate adjustment was included for body mass index (BMI), age, sex, and the top five principal components of genetic ancestry. The analysis was restricted to participants in the White British ancestry subset (UKB Field ID: 22006).

#### Association With HCC

For each candidate variant, we computed the simple minor allele frequencies (MAFs) in cases and controls from all four cohorts. The association between each candidate variant and HCC was then quantified through multivariate logistic regression. All associations were adjusted for age, sex, and the top principal components of genetic ancestry. However, there were minor differences by data set, which are outlined in Supporting Table S3. We did not control for established HCC risk variants (i.e., rs738409 in *PNPLA3*, rs58542926 in *TM6SF2,* and rs72613567 in *HSD17B13*) because these were all in linkage equilibrium with the candidate variants considered (i.e., *R* ^2^ < 0.001). The exception to this was rs187429064 where we included adjustment for the rs58542926 genotype out of prudence, given that both variants lie in *TM6SF2*. All associations were calculated under an additive genetic model, with two‐tailed *P* values presented.

We then performed a fixed‐effect meta‐analysis to determine a pooled effect size across studies, using the METAL software package.^(^ ^22^ ^)^ Two pooled effect sizes were calculated. First, a pooled effect size specific to cirrhosis controls (i.e., UKB controls 1+Dresden controls 1+STOP‐HCV). Second, an overall effect size specific to population controls (i.e., UKB controls 1+FinnGen). All meta‐analyses were weighted according to the effective sample size, defined according to the formula 4/(1/number of cases + 1/number of controls). A Bonferroni‐corrected *P* < 0.0125 was used to judge statistical significance.

Associations were expressed either in terms of log odds ratio (LOR) or odds ratios (ORs), where the latter is simply the exponent of the LOR. In graphical figures, we present associations in terms of their LORs because these are symmetrical around the null and thus allow one to visually compare magnitude of associations for variants that affect risk in opposing directions. Various polygenic risk scores were also created, and their association with HCC was quantified (see Supporting Materials Appendix B).

## Results

### Association With Liver Fat Content

**FIG. 1:**
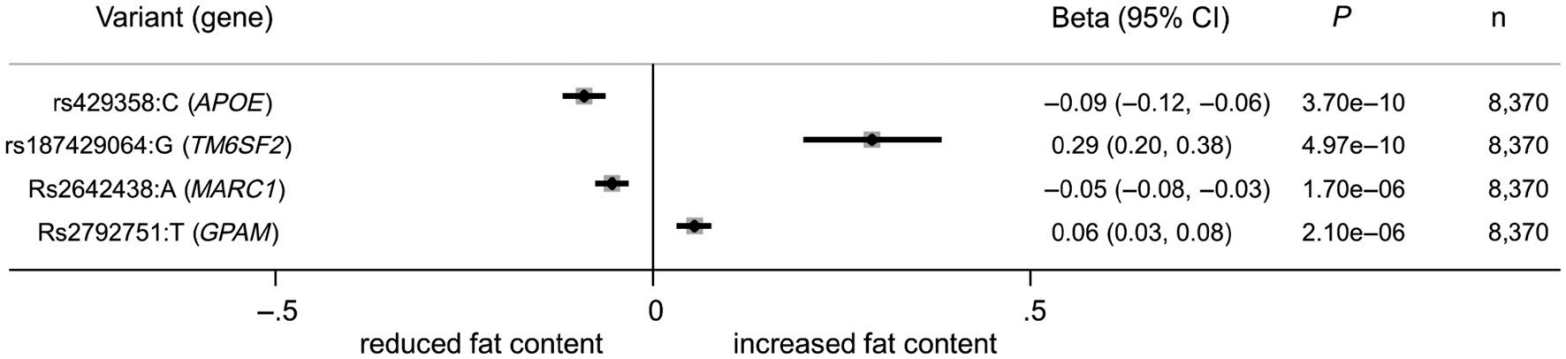
Association of candidate variants with liver fat content in the UKB study.

All four variants were strongly associated with liver fat content, with *P* values ranging from 2.1 × 10^−6^ to 3.7 × 10^−10^ (see Fig. 1). Two of the four variants were associated with reduced liver fat content (rs429358:C in *APOE* and rs2642438:A in *MARC1*), whereas two variants were associated with increased liver fat content (rs2792751:T in *GPAM* and rs187429064:G in *TM6SF2*). The rs187429064:G variant exhibited the strongest effect size (beta, 0.29), followed by rs429358:C (beta, −0.09), then rs279275:T (beta, 0.06), and then rs2642438:A (beta, −0.05).

### Case‐Control Data

In total, the four case‐control data sets included 2,070 HCC cases and 4,121 cirrhosis controls. Over half the cases were alcohol‐related HCCs from the Dresden study (n = 1,289), and 149 were hepatitis C‐related HCCs from the STOP‐HCV study. There were 366 HCC cases identified from the UKB study. Of these, we estimate that 153 (43%) were related to NAFLD, 115 (31%) related to ArLD, and 29 (9%) related to viral hepatitis. Cases were largely men (73%‐91%), with a mean age ranging from 60 to 69 years depending on the study (Table 1).

**TABLE 1:** Summary of the case‐control data sets used in this study

Data Source	Cohorts	Characteristic			Minor Allele Frequency (%)			
		Number*	Mean Age, Years	Sex (% Men)	rs429358 C *(APOE)*	rs2792751 T *(GPAM)*	rs2642438 A *(MARC1)*	rs187429064 G *(TM6SF2)*
UKB	Cases: HCC	366	62.1	77	10.8	29.1	25.6	3.6
	Controls 1: hospital admission for cirrhosis without HCC	2,536	59.3	63	13.9	28.2	28.1	1.3
	Controls 2: all UKB participants without HCC^†^	3,49,018	57.5	47	15.6	27.4	29.7	1.1
FinnGen	Cases: primary liver cancer	266	68.9	74	12.4	33.0	25.8	12.5
	Controls: all participants without primary liver cancer^†^	1,76,633	NK	NK	18.5	31.9	28.4	5.1
Dresden alcohol cohort	Cases: HCC and alcohol‐related cirrhosis	1,289	65.0	91	8.9	33.6	25.4	2.4
	Controls 1: alcohol‐related cirrhosis without HCC	894	57.1	75	11.8	32.0	26.4	1.3
	Controls 2: heavy drinkers with neither significant liver disease nor HCC	128	60.6	70	14.6	32.2	31.1	0.8
STOP‐HCV	Cases: HCC and hepatitis C‐related cirrhosis	149	60.3	73	9.4	28.9	24.8	1.7
	Controls: hepatitis C‐related cirrhosis without HCC	691	55.8	77	13.0	29.9	29.7	0.9

* Number of cases indicated here may differ from the number used in regression analyses due to missing data for genotype and/or age, and/or sex.

^†^ Control group largely comprises individuals with no history of liver disease.

Of the cirrhosis controls, 691 were from STOP‐HCV, 2,536 from the UKB, and 894 from Dresden. Cirrhosis controls were again predominantly men (63%‐77%) but were younger than HCC cases (mean age ranging from 55 to 59 years). The UKB and FinnGen non‐liver disease control groups comprised 349,018 and 176,633 FinnGen individuals, respectively (Table 1). Of the 128 noncirrhotic controls from the Dresden cohort, 50%, 40%, and 10% were estimated to be at Metavir stage F0, F1‐2, and F3, respectively, based on AST‐adapted liver stiffness cutoffs.

In the STOP‐HCV cohort, about one fifth (19.6%) had achieved a hepatitis C sustained viral response at the time of study enrollment. This proportion was comparable for HCC cases (20.8%) and cirrhosis controls (19.4%).

### Association With HCC

#### *APOE* (rs429358)

**FIG. 2:**
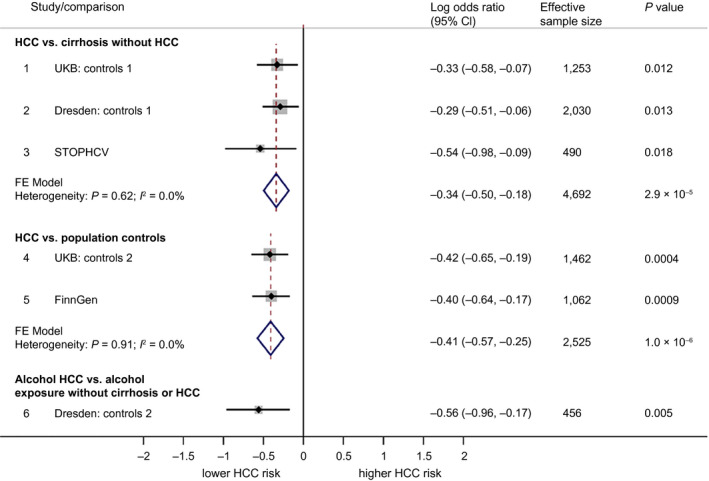
Forest plot showing association between rs429358:C (*APOE*) and HCC. Associations are broken down into the following three categories: 1) comparing HCC to cirrhosis controls without HCC, 2) comparing HCC to population controls (who for the most part will not have liver disease), and 3) comparing alcohol HCC to individuals with an alcohol exposure but without cirrhosis or HCC. Associations are presented in terms of the LOR. An LOR of 0 indicates that the frequency of rs429358:C is the same for cases as for controls. LORs were calculated using logistic regression under an additive genetic model. Pooled effects are based on fixed‐effect meta‐analysis, weighted by effective sample size.

The *APOE* rs429358:C allele was consistently less frequent in cases versus controls across all data sets. For example, 10.8% in UKB cases versus 13.9% in non‐HCC controls (Table 1). In multivariate regression, rs429358:C was independently associated with a reduced HCC risk across all comparisons and data sets. The pooled OR for each copy of the rs429358:C allele was 0.71 (95% confidence interval [CI], 0.61‐0.84; *P* = 2.9 × 10^−5^) against cirrhosis controls and 0.66 (95% CI, 0.57‐0.78; *P* = 1.0 × 10^−6^) against population controls (see Fig. 2).

#### *GPAM* (rs2792751)

**FIG. 3:**
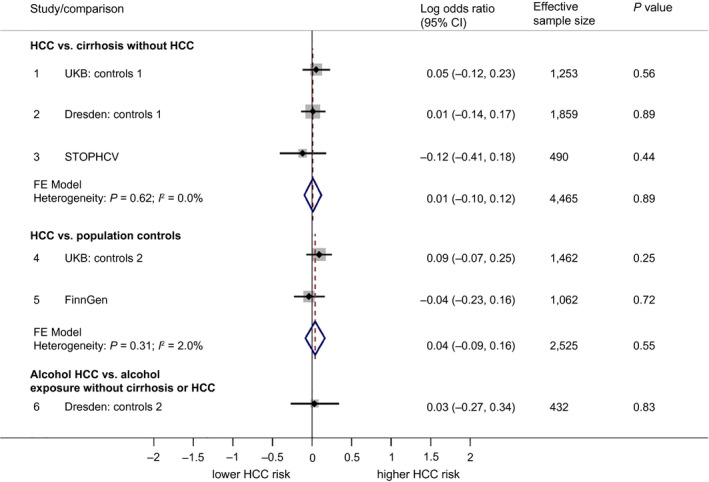
Forest plot showing association between rs2792751:T (*GPAM*) and HCC. Associations are broken down into the following three categories: 1) comparing HCC to cirrhosis controls without HCC, 2) comparing HCC to population controls (who for the most part will not have liver disease), and 3) comparing alcohol HCC to individuals with an alcohol exposure but without cirrhosis or HCC. Associations are presented in terms of the LOR. An LOR of 0 indicates that the frequency of rs2792751:T is the same for cases as for controls. LORs were calculated using logistic regression under an additive genetic model. Pooled effects are based on fixed‐effect meta‐analysis, weighted by effective sample size.

The *GPAM* rs2792751:T allele was generally higher in HCC cases versus controls. However, the differences were modest; for example, 33.6% in Dresden HCC cases versus 32.0% in cirrhosis controls. In multivariate regression, the associations were not significant. The pooled OR for each copy of the rs2792 T allele was 1.01 (95% CI, 0.90‐1.13; *P* = 0.89) against cirrhosis controls and 1.04 (95% CI, 0.91‐1.17; *P* = 0.55) against population controls (see Fig. 3).

#### *MARC1* (rs2642438)

**FIG. 4:**
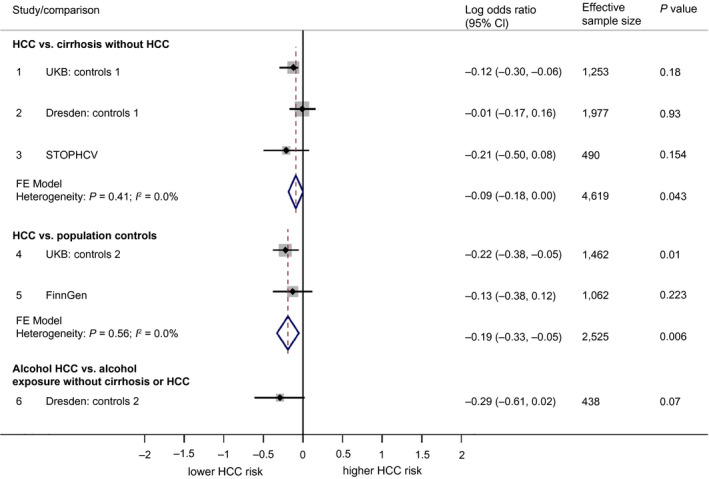
Forest plot showing association between rs2642438:A (*MARC1*) and HCC. Associations are broken down into the following three categories: 1) comparing HCC to cirrhosis controls without HCC, 2) comparing HCC to population controls (who for the most part will not have liver disease), and 3) comparing alcohol HCC to individuals with an alcohol exposure but without cirrhosis or HCC. Associations are presented in terms of the LOR. An LOR of 0 indicates that the frequency of rs2642438:A is the same for cases as for controls. LORs were calculated using logistic regression under an additive genetic model. Pooled effects are based on fixed‐effect meta‐analysis, weighted by effective sample size.

The rs2642438:A variant in *MARC1* was consistently less frequent in HCC cases versus controls. For example, 24.8% for HCC cases in STOP‐HCV versus 29.7% in controls. In regression analysis, the association was relatively weak. The pooled OR for each copy of the rs2642438:A allele was 0.91 (95% CI, 0.84‐1.00; *P* = 0.043) against cirrhosis controls and 0.83 (95% CI, 0.72‐0.95; *P* = 0.006) against population controls (see Fig. 4).

#### *TM6SF2* (rs187429064)

**FIG. 5:**
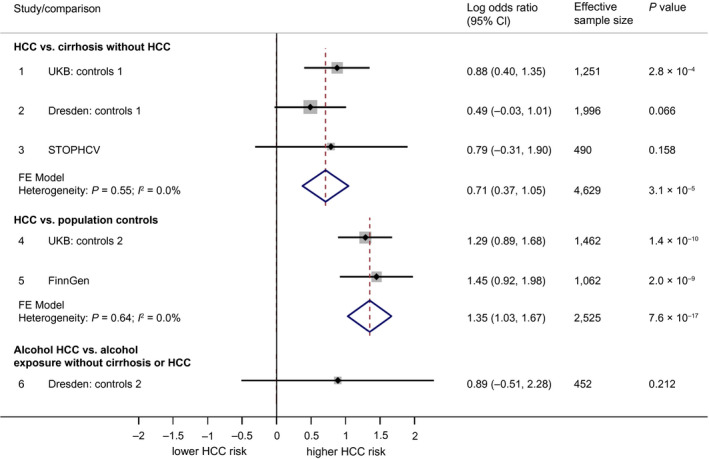
Forest plot showing association between rs187429064:G (*TM6SF2*) and HCC. Associations are broken down into the following three categories: 1) comparing HCC to cirrhosis controls without HCC, 2) comparing HCC to population controls (who for the most part will not have liver disease), and 3) comparing alcohol HCC to individuals with an alcohol exposure but without cirrhosis or HCC. Associations are presented in terms of the LOR. An LOR of 0 indicates that the frequency of rs187429064:G is the same for cases as for controls. LORs were calculated using logistic regression under an additive genetic model. Pooled effects are based on fixed‐effect meta‐analysis, weighted by effective sample size.

The rs187429064:G variant in *TM6SF2* was consistently higher in cases versus controls. For example, 3.6% in UKB cases versus 1.1% in all UKB controls without HCC. In regression analysis, rs187429064:G was associated with a higher HCC risk across all comparison and data sets. The pooled OR for each copy of the rs187429064 G allele was 2.03 (95% CI, 1.45‐2.86; *P* = 3.1 × 10^−6^) against cirrhosis controls and 3.86 (95% CI, 2.80‐5.31; *P* = 7.6 × 10^−17^) against population controls (see Fig. 5). Genotypic ORs were also calculated for each candidate variant and were generally consistent with allelic ORs (see Supporting Figs. S1‐S4).

### *APOE* Exploratory Analysis

We performed three exploratory analyses to better understand the association between rs429358 in *APOE* and HCC. First, we tested for interaction between rs429358:C and polymorphisms in the low‐density lipoprotein receptor (*LDLR*) gene. From the UKB genetic data set, 144 polymorphisms in *LDLR* with a MAF > 1% that did not violate the assumption of Hardy‐Weinberg equilibrium were extracted. Rs429358:C epistasis with each of these 144 polymorphisms was assessed in our UKB case‐control data set. The lead variant from this scan was rs1569372 (Supporting Figs. S5 and S6), indicating the rs429358:C association with HCC was stronger in the presence of the rs1569372 G allele (*P* for interaction = 0.020). However, this interaction did not replicate in the Dresden and STOP‐HCV cohorts (see Supporting Fig. S7).

Second, to generate insight into the underlying biological mechanisms, we assessed attenuation in the rs429358:C–HCC association following adjustment for selected biomarkers and/or detailed confounding factors (UKB data set only). The factors considered in this analysis were total cholesterol (Field ID: 30690), high‐density lipoprotein cholesterol (HDL‐C) (Field ID: 30760), low‐density lipoprotein cholesterol (LDL‐C) (Field ID: 30780), lipoprotein A (Field ID: 30790), tyriglycerides (Field ID: 30870), APOA (Field ID: 30630), APOB (Field ID: 30640), glycated hemoglobin A1c (Field ID: 30750), BMI (Field ID: 21001), C‐reactive protein (Field ID: 30710), statin therapy (Field ID: 20003), telomere length (Field ID: 22191), and current tobacco smoking (Field ID: 20116). All factors/biomarkers were measured at the time of UKB study recruitment. Attenuation in the rs429358–HCC association was modest/negligible for the majority of factors. The strongest attenuation occurred in relation to lipoprotein A, where the LOR attenuated to −0.19 (95% CI, −0.49 to 0.10; *P* = 0.20) (Supporting Figs. S8 and S9).

Third, using haplotype data from the UKB genetic data set, we characterized participants according to ε2, ε3, and ε4 APOE alleles^(^ ^23^ ^)^ and assessed the association of these alleles with HCC. Relative to ε3‐ε3, the ε4‐ε4 haplotype was associated with the greatest effect size (OR, 0.61; 95% CI, −0.23 to 0.64; *P* = 0.30), followed by ε3‐ε4 (OR, 0.68; 95% CI, 0.51‐0.93; *P* = 0.015), followed by ε2‐ε4 (OR, 0.72; 95% CI, 0.32‐1.62; *P* = 0.42) (Supporting Figs. S10 and S11). In this vein, we also assessed the relationship between rs7412 in *APOE* and case‐control status directly and did not find any significant association (Supporting Fig. S12).

## Discussion

Although HCC is a leading cause of cancer mortality, the genetic factors that predispose individuals to this outcome are not fully understood. In this study, we highlight the importance of two (hitherto unrecognized) germline genetic polymorphisms with respect to HCC risk. These variants were rs429358:C in *APOE*, which associates with a lower risk of HCC, and rs187429064:G in *TM6SF2*, which associates with a higher HCC risk. Each variant exhibited a consistent trend across all data sets, and their direction of association with HCC mirrors their direction of association with hepatic fat content.

The relevance of rs2642438:A allele in *MARC1* is more equivocal. Although this variant was less frequent among HCC cases in some data sets, the effect size in the pooled analysis was relatively modest and did not quite reach statistical significant when comparing against cirrhosis controls (*P* = 0.02). Conversely, the association between rs2792751:T in *GPAM* and HCC was consistently close to the null, suggesting it is not a relevant risk factor for HCC, despite its strong association with hepatic fat content.

APOE is found at the surface of lipoprotein particles and plays a pivotal role in lipid transport to and from the liver.^(^ ^23^ ^)^ Rs429358:C is a coding variant that leads to a cysteine to arginine replacement at position 112/317 of the APOE protein. This polymorphism is notorious for its adverse effect on Alzheimer’s disease (AD), yet it also modulates the risk of cardiovascular disease (CVD) and other health outcomes.^(^ ^24^, ^25^ ^)^ Previous studies have shown that rs429358:C is associated with a reduced risk of cirrhosis in HCV,^(^ ^25^ ^)^ NAFLD,^(^ ^8^ ^)^ and in mixed‐etiology population cohorts.^(^ ^26^, ^27^ ^)^ Our results extend this narrative by demonstrating that rs429358:C is also associated with a reduced risk of HCC in HCV cirrhosis, alcohol‐related cirrhosis, and in a mixed‐etiology cirrhosis cohort (i.e., UKB). The persistence of this association when comparing against cirrhosis controls strongly implies that *APOE* has a direct role in liver carcinogenesis and does not simply reduce HCC by altering progression to cirrhosis. Nevertheless, the specifics of what this direct role could be are unclear. It has been known for some time, that rs429358:C is associated with higher serum cholesterol levels.^(^ ^28^ ^)^ More recently, Qin et al.^(^ ^29^ ^)^ demonstrated that inducing higher serum cholesterol in mice (both through diet and genetic disruption of the *ApoE* gene) leads to enhanced HCC suppression after injection with a chemical carcinogen. The authors indicate that higher serum cholesterol may increase the cancer immunosurveillance activity of natural killer cells. Consistent with this, they also reported a correlation between the serum cholesterol and natural killer cell activity in human HCC tissue. Thus, an obvious question is whether the rs429358–HCC association is merely a corollary for differences in serum cholesterol levels according to rs429358 genotype. However, there might be additional molecular aspects involved because only modest levels of attenuation in the rs429358–HCC association were observed when adjusting for HLD‐C, LDL‐C, total cholesterol, and broader measures of dyslipidemia. We also investigated if the rs429358–HCC association varied according to polymorphisms in *LDLR* because interaction between loci in these genes has been observed for AD and CVD^(^ ^30^, ^31^, ^32^ ^)^ and because APOE is a ligand for the LDLR.^(^ ^33^ ^)^ Our results from this analysis suggest that the rs429358–HCC association may be stronger for carriers of the rs73015034:C allele in LDLR. However, further studies are needed to explore this conceivable functional link in greater detail.

We also investigated the association between the ε2, ε3, and ε4 APOE alleles and HCC, where ε2, ε3, and ε4 are determined by genotype at rs429358 and rs7412 loci. As expected, this analysis showed that the ε4 allele, defined by the presence of the rs429358:C and rs7412:C allele on the same copy of chromosome 19, was less frequent in HCC cases versus controls. This begs the question of whether it is the ε4 haplotype (i.e., combination of rs429358:C and rs7412:C on the same chromosome) or rs429358:C alone that drives the protective effect. However, because the two are effectively synonymous, i.e., the overwhelming majority of individuals with rs429358:C also carry rs7412:C on that same chromosome, it is difficult to disentangle the effect of one from the other.

This study also identifies a missense variant in *TM6SF2* (rs187429064:G) as being associated with HCC among patients with cirrhosis. *TM6SF2* has been widely studied in connection to rs58542926:T, another missense variant that is itself associated with HCC and also liver cirrhosis^(^ ^4^, ^6^, ^15^, ^34^ ^)^ but interestingly protects from CVDs.^(^ ^35^ ^)^ Previous work indicates that loss of TM6SF2 function increases hepatocyte fat content by reducing APOB secretion.^(^ ^36^ ^)^ This is consistent with a study by Pelusi et al.^(^ ^37^ ^)^ showing that individuals with rare pathogenic variants in *APOB* are at increased risk of HCC. The present data therefore corroborate the importance of *TM6SF2* in relation to HCC oncogenesis. The association we observed between the rs187429064 locus and HCC cannot be explained in terms of confounding by the rs58542926 genotype because our regression models included adjustment for rs58542926, and in any case, rs58542926 and rs187429064 genotype status are not correlated with one another in Europeans (*R* ^2^ = 0.0009). At the protein level, rs187429064:G results in a lysine to arginine substitution at position 156/377. Although the frequency of this variant is relatively rare in Europeans (allele frequency ~1%), it can vary widely from one population to another. For example, in the FinnGen cohort, rs187429064:G has an allele frequency of 5.1%, which is comparable to rs58542926:T.

Our study has a number of strengths. First, despite HCC being a relatively rare outcome, we have succeeded in assembling a large sample size with over 2,000 HCC cases. This has enabled us to generate precise effect‐size estimates for each variant. A second strength is our inclusion of both cirrhosis and population control groups, each of which complements the other. Third, we were able to draw on data from a variety of cohorts and etiologies. This affords us a level of confidence regarding the generalizability of our findings to other settings and patient groups.

A limitation of this study is that we did not have access to an equivalent case‐control data set for NAFLD, and thus we cannot say if our findings extend to this etiology specifically. Second, our base case analysis included adjustment for only age, sex, and principal components of genetic ancestry. Ideally, we would have adjusted for smoking, BMI, diabetes, and statin use, but these covariates were missing for a substantial proportion of patients in the STOP‐HCV and Dresden cohorts. However, we did perform an analysis to assess attenuation of the rs429358–HCC association following adjustment for statin use, smoking, BMI and other covariates. The level of attenuation observed was marginal, suggesting that the rs429358–HCC association cannot be explained in terms of simple confounding. Another limitation relates specifically to the FinnGen data, where the outcome event considered was primary liver cancer as opposed to HCC. However, we do not think this is likely to have exerted much bias on our results given that HCC accounts for the large majority of primary liver cancer cases. Finally, this study was restricted to individuals of Caucasian ethnicity in order to circumvent confounding by population structure. We do not know if these variants are relevant to HCC risk for individuals in other ethnic groups.

Overall, this study has helped to further elucidate the genetic background to HCC by showing that two variants, one in *APOE* and a second missense variant in *TM6SF2*, are associated with HCC across a variety of data sets and etiologies. These associations have not previously been identified or recognized hitherto. Also, despite a strong association with liver fat content, the rs2792751:T missense variant in *GPAM* does not appear to influence HCC risk. These findings will help fine‐tune emerging HCC risk stratification tools and allow greater insight into currently unknown molecular aspects of HCC oncogenesis.

## Supporting information

Supplementary Material
