# Supplementary material for: The rs429358 Locus in Apolipoprotein E Is Associated With Hepatocellular Carcinoma in Patients With Cirrhosis
Source: Hepatol Commun. 2021 Dec 27;6(5):1213–26. doi: 10.1002/hep4.1886 (PMC9035556; doi:10.1002/hep4.1886)
Supplement: Supplementary file 1 — Supplementary Material [file HEP4-6-1213-s001.docx]

**The rs429358 loci in *APOE* is associated with hepatocellular carcinoma in patients with cirrhosis.**

Hamish Innes, Hans Dieter Nischalke, Indra Neil Guha, Karl Heinz Weiss, Will Irving, Daniel Gotthardt, Eleanor Barnes, Janett Fischer, Azim Ansari, Jonas Rosendahl, Shang-Kuan L, Astrid Marot, Vincent Pedergnana, Markus Casper, Jennifer Benselin, Frank Lammert, John McLauchlan, Philip L Lutz, Victoria Hamill, Sebastian Mueller, Joanne R Morling, Georg Semmler, Florian Eye, Johann von Felden, Alexander Link, Arndt Vogel, Jens U Marquardt, Stefan Sulk, Jonel Trebicka, Luca Valenti, Christian Datz, Thomas Reiberger, Clemens Schafmayer, Thomas Berg, Pierre Deltenre*****, Jochen Hampe*****, Felix Stickel*****, Stephan Buch*****.

^*^PD, JH, FS and SB have contributed equally to this work and therefore share senior authorship

**Table of contents**

**Supplementary Figure 1………………………………………………………….…..3**

**Supplementary Figure 2………………………………………………………….…..4**

**Supplementary Figure 3………………………………………………………….…..5**

**Supplementary Figure 4………………………………………………………….…..6**

**Supplementary Figure 5………………………………………………………….…..7**

**Supplementary Figure 6………………………………………………………….…..8**

**Supplementary Figure 7………………………………………………………….…..9**

**Supplementary Figure 8………………………………………………………….…10**

**Supplementary Figure 9………………………………………………………….…11**

**Supplementary Figure 10…………………………………………………………...12**

**Supplementary Figure 11…………………………………………………………...13**

**Supplementary Figure 12…………………………………………………………...14**

**Supplementary Table 1……………………………………………………………...15**

**Supplementary Table 2……………………………………………………………...16**

**Supplementary Table 3……………………………………………………………...17**

**Appendix A………………………………………………………………18-19**

**Appendix B………………………………………………………………19-22**


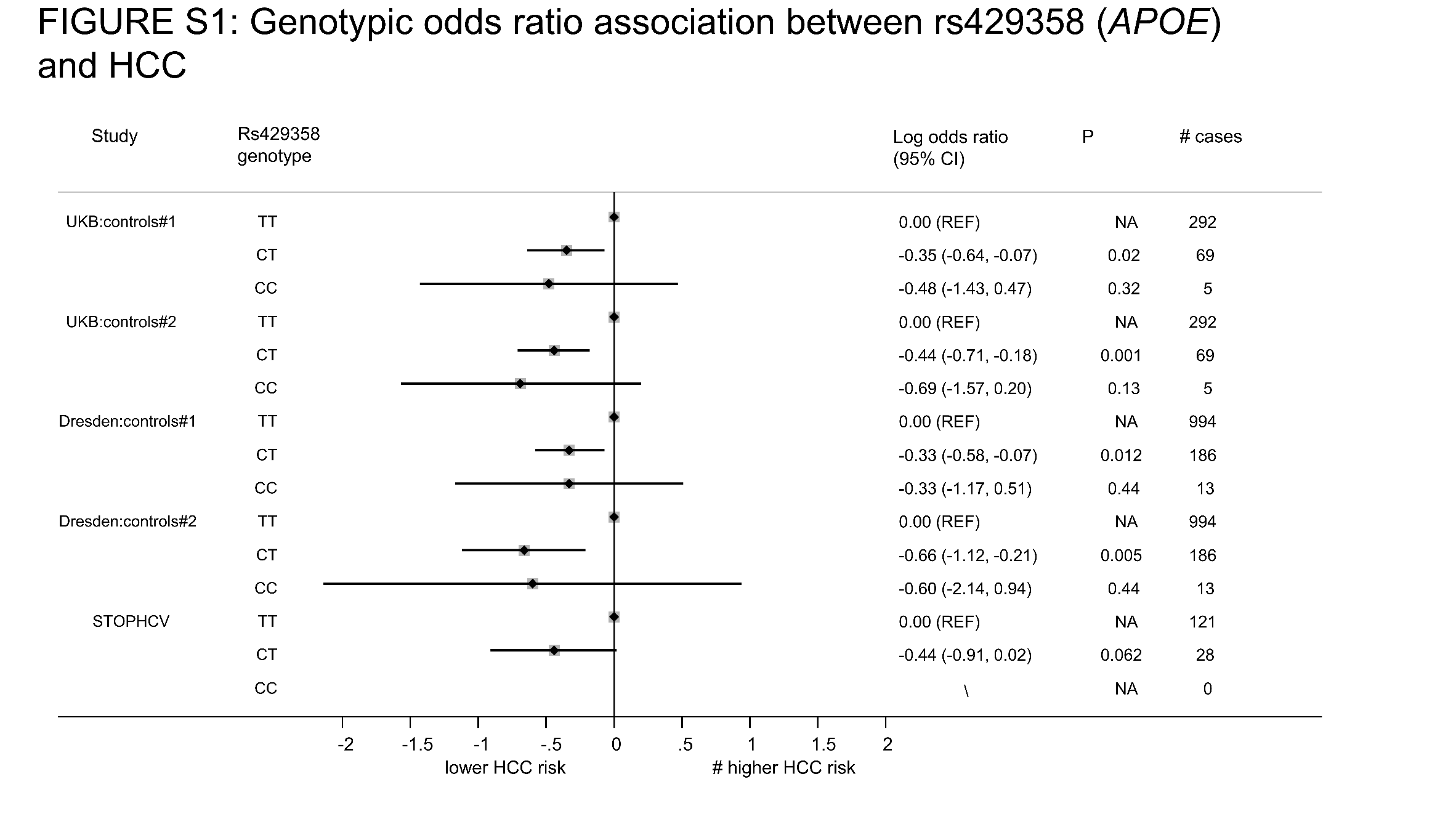


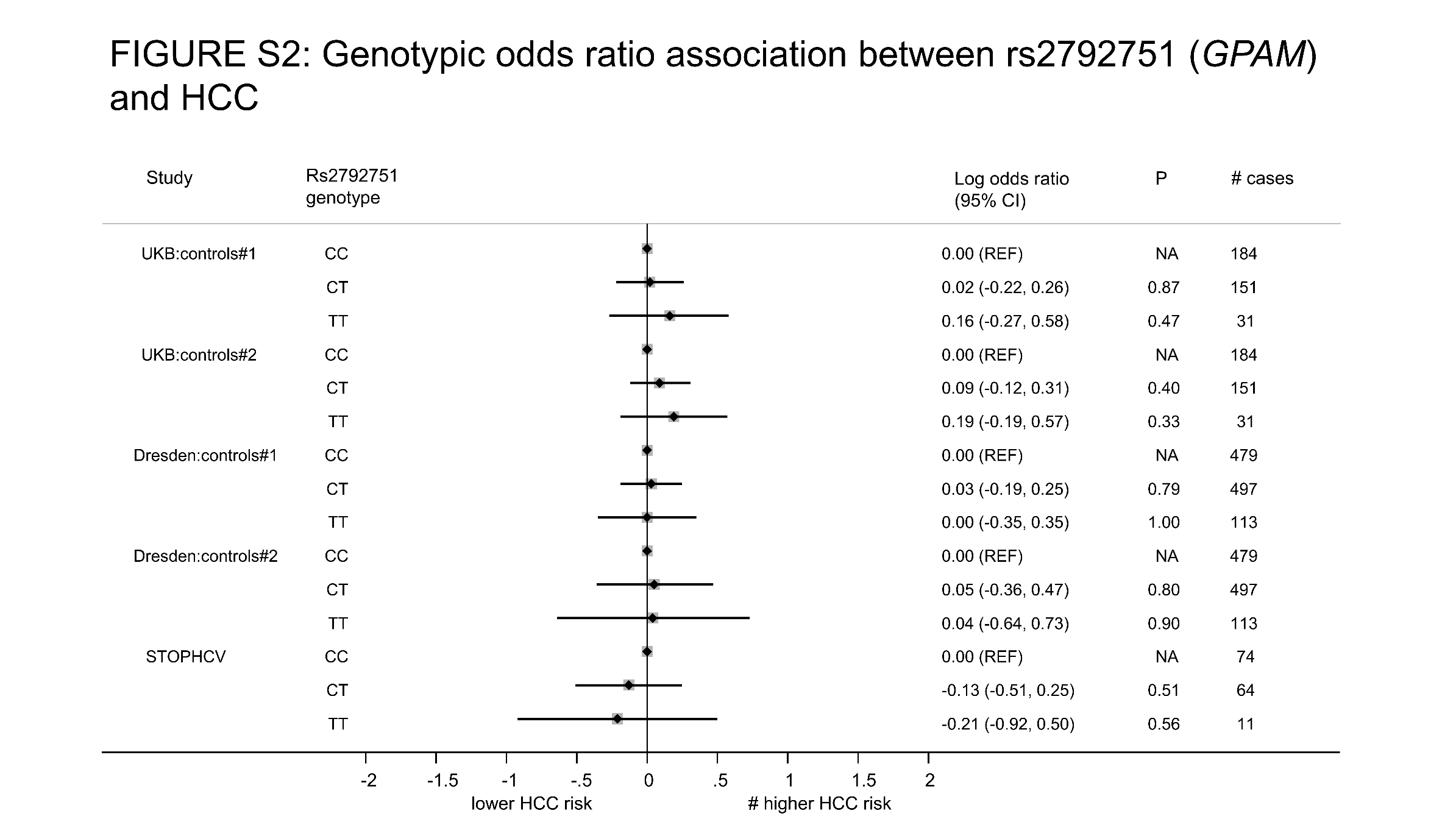


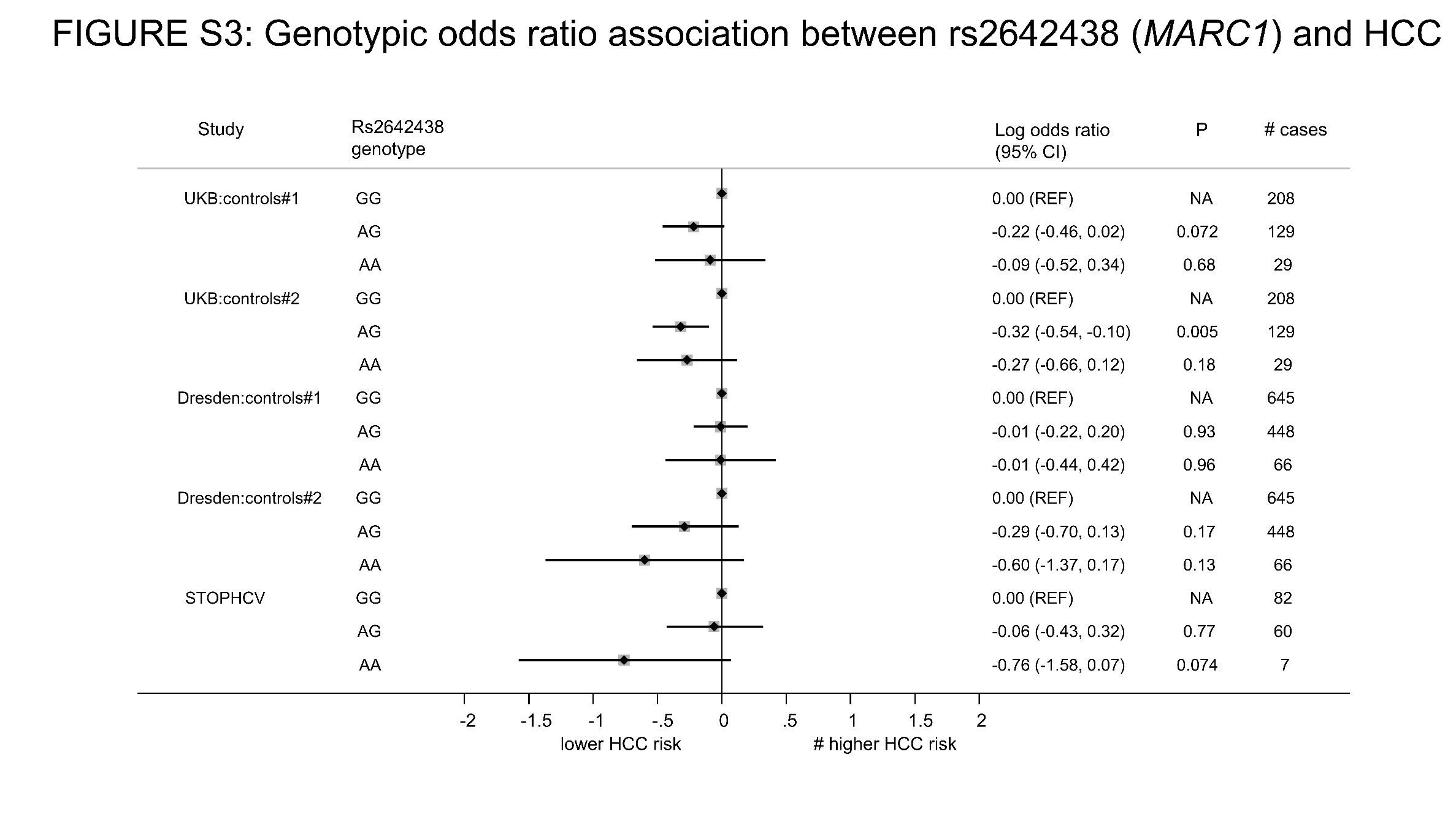


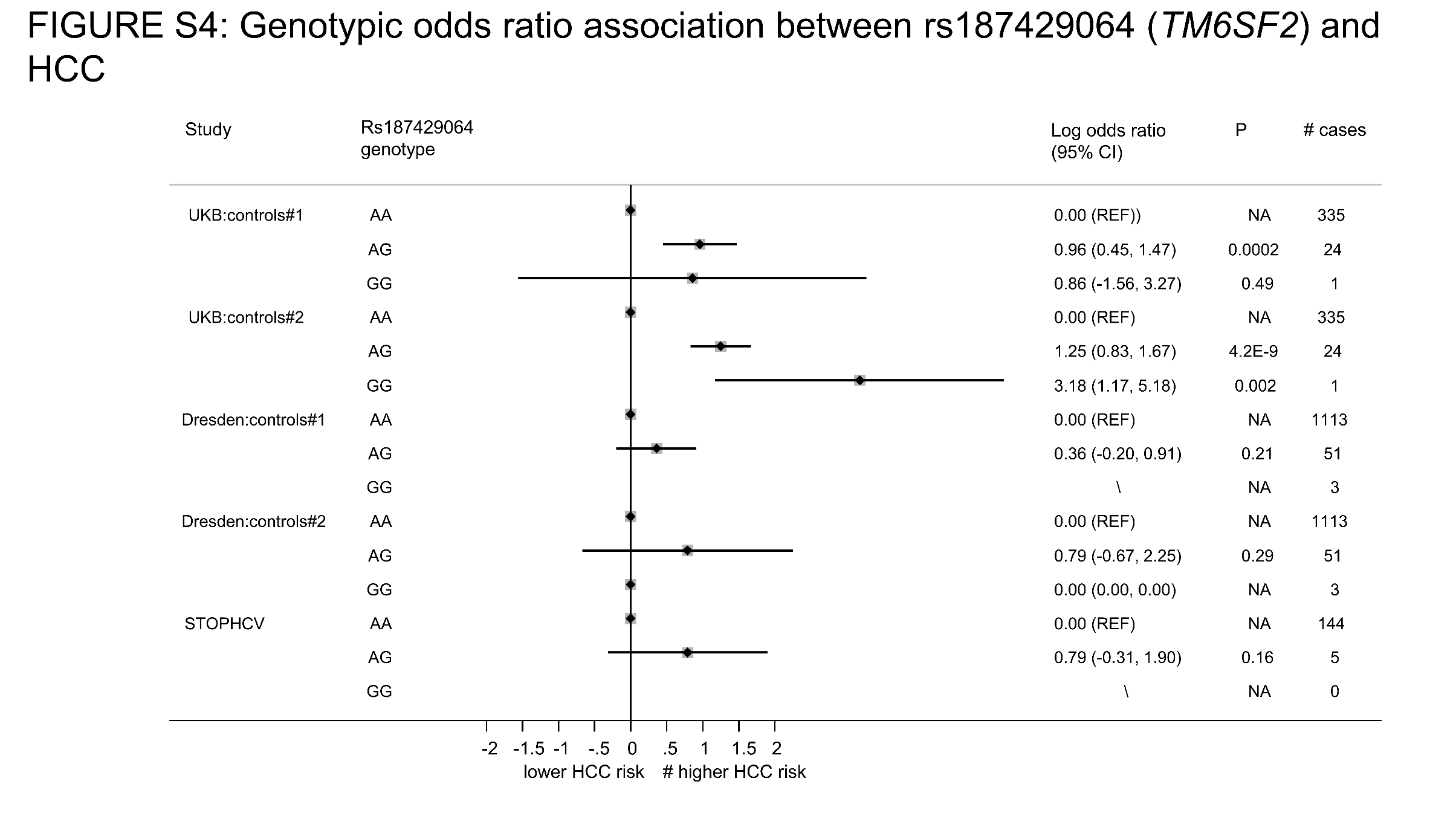


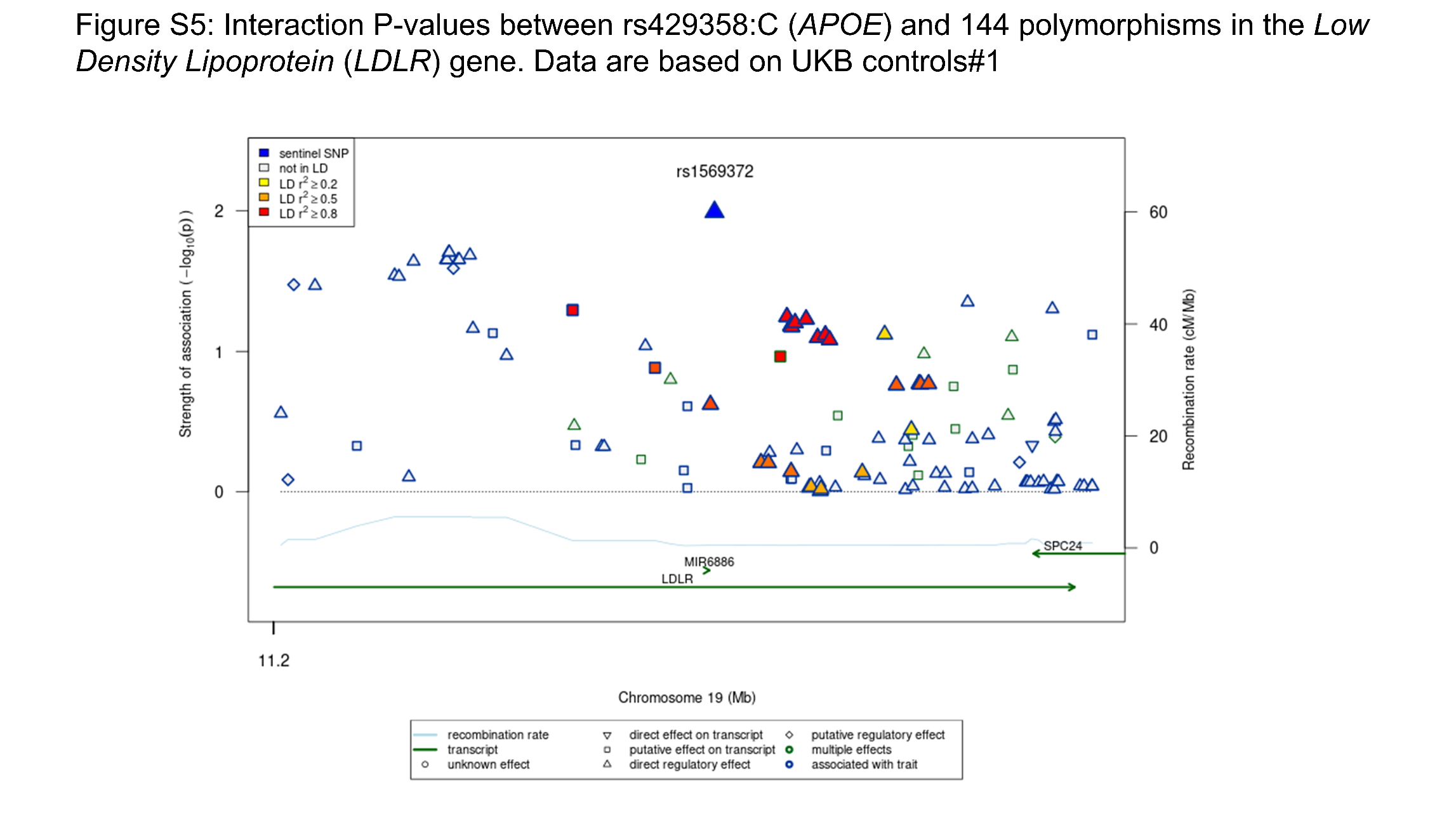


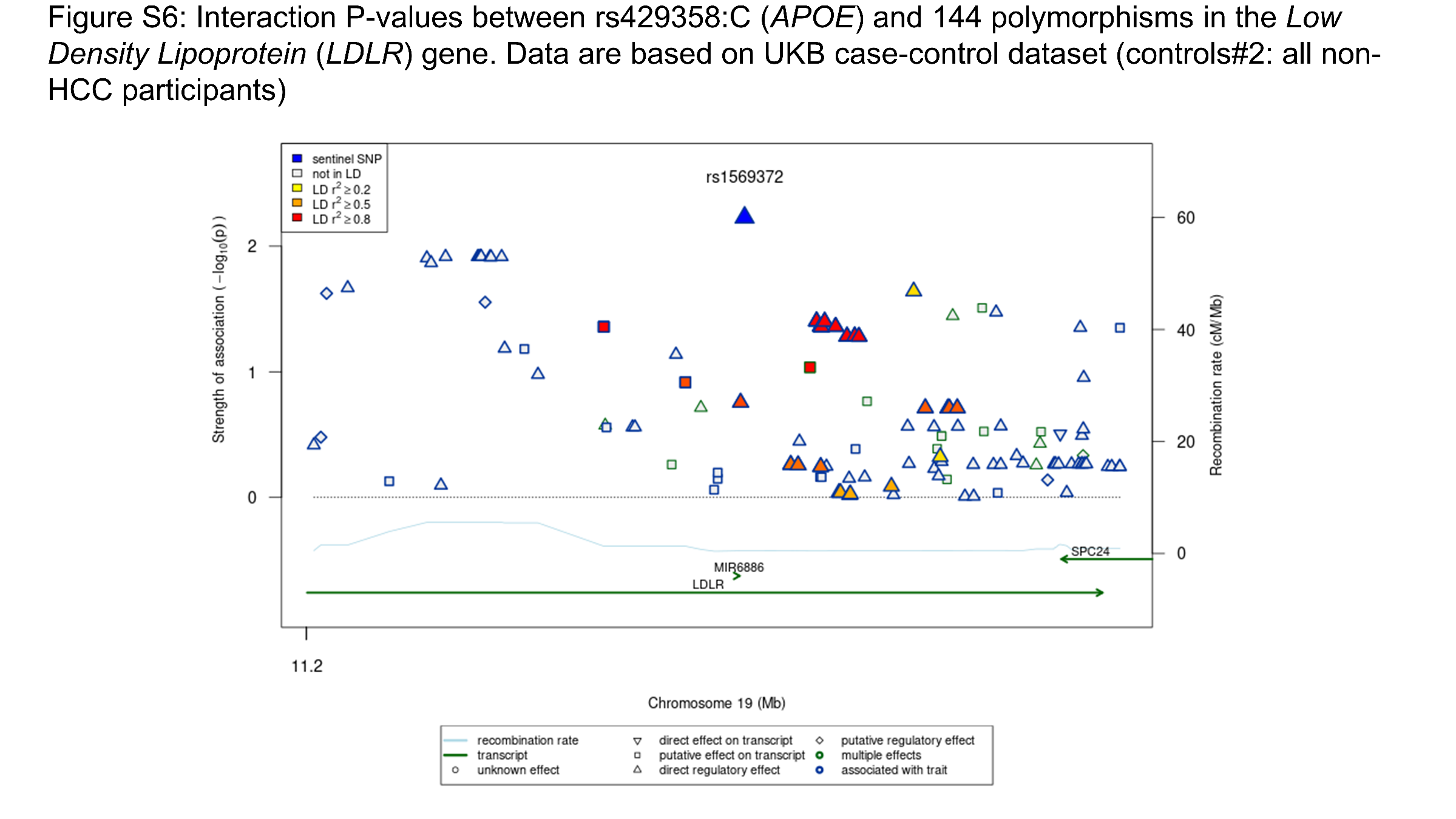


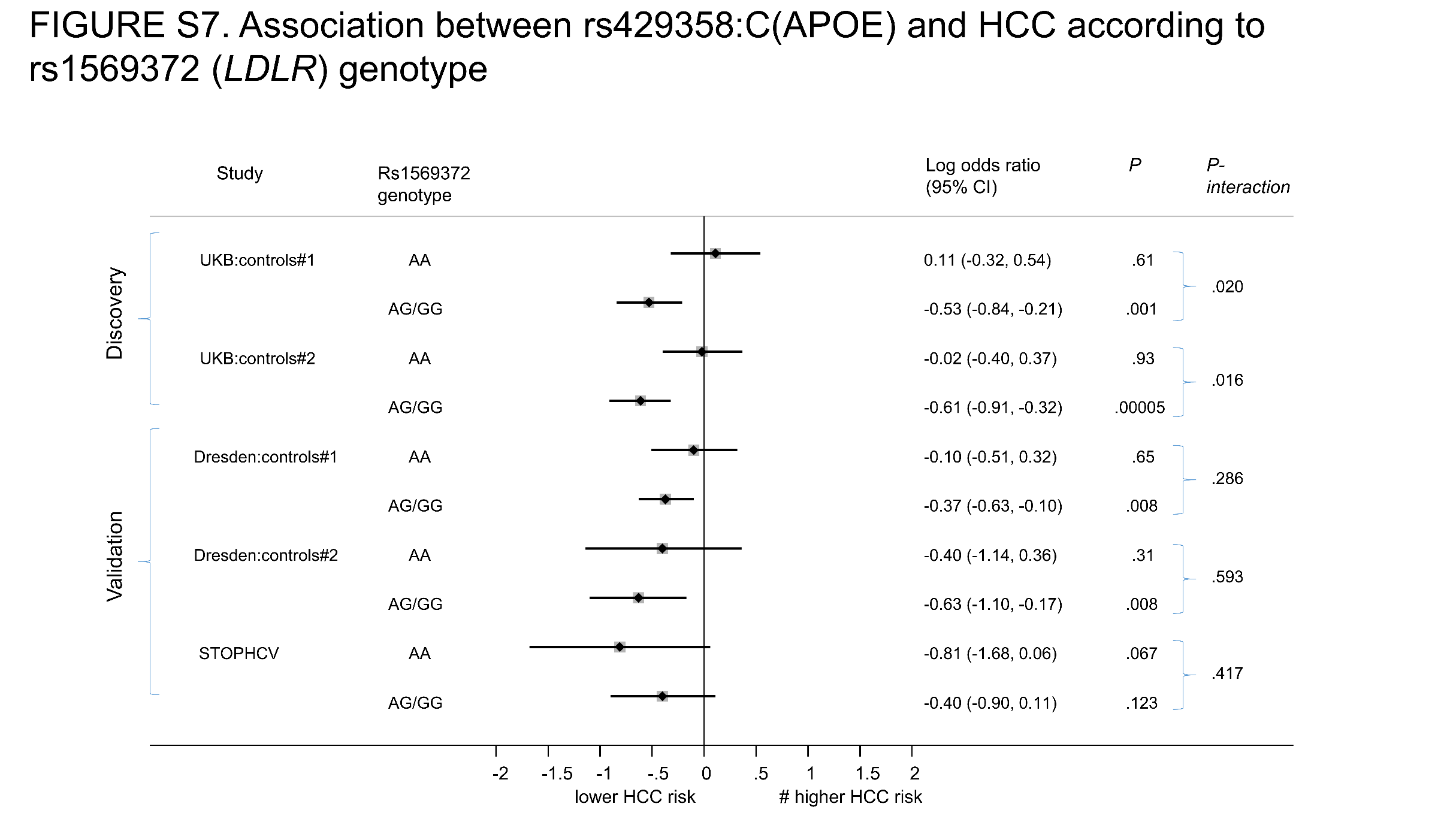


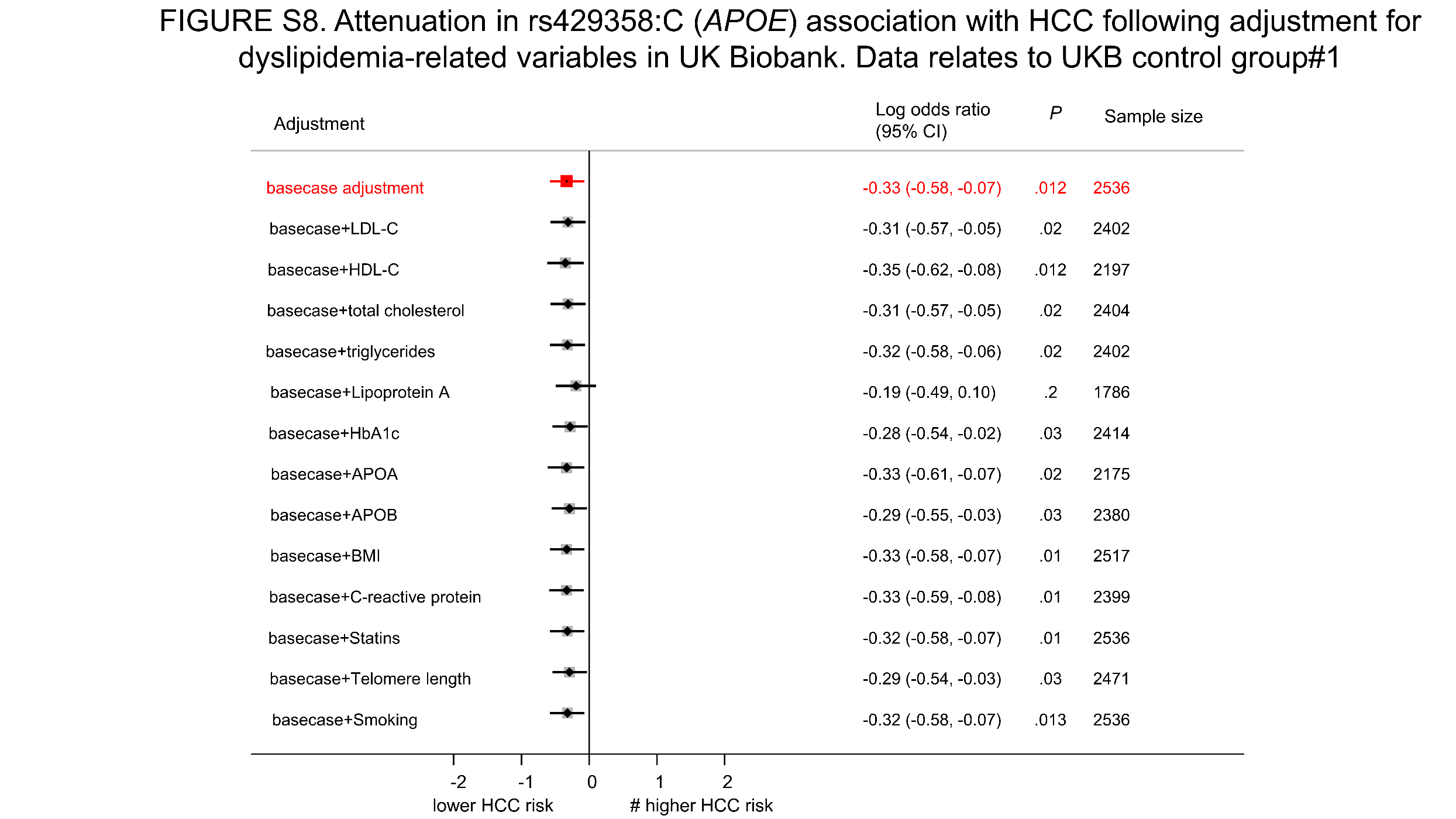


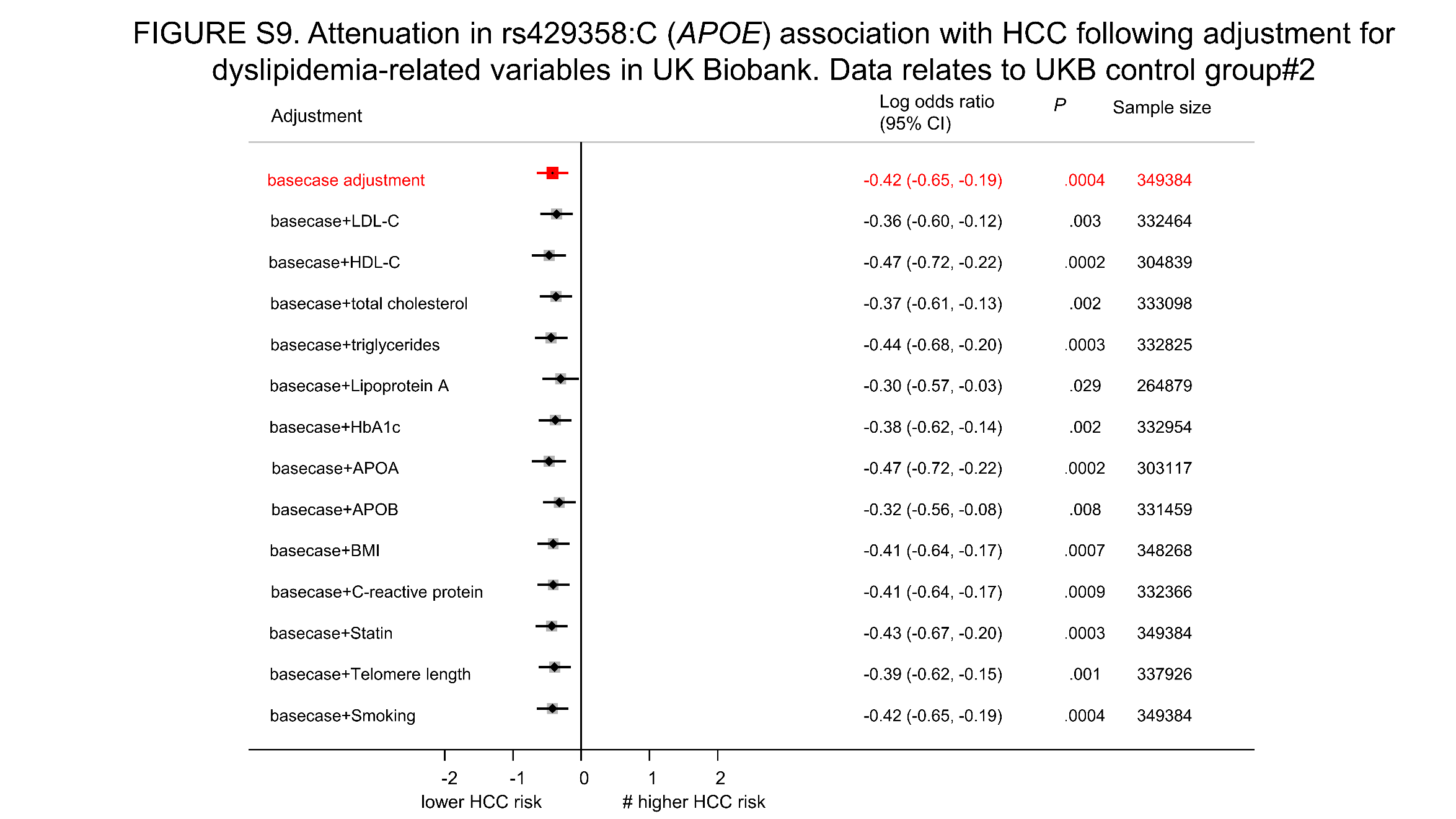


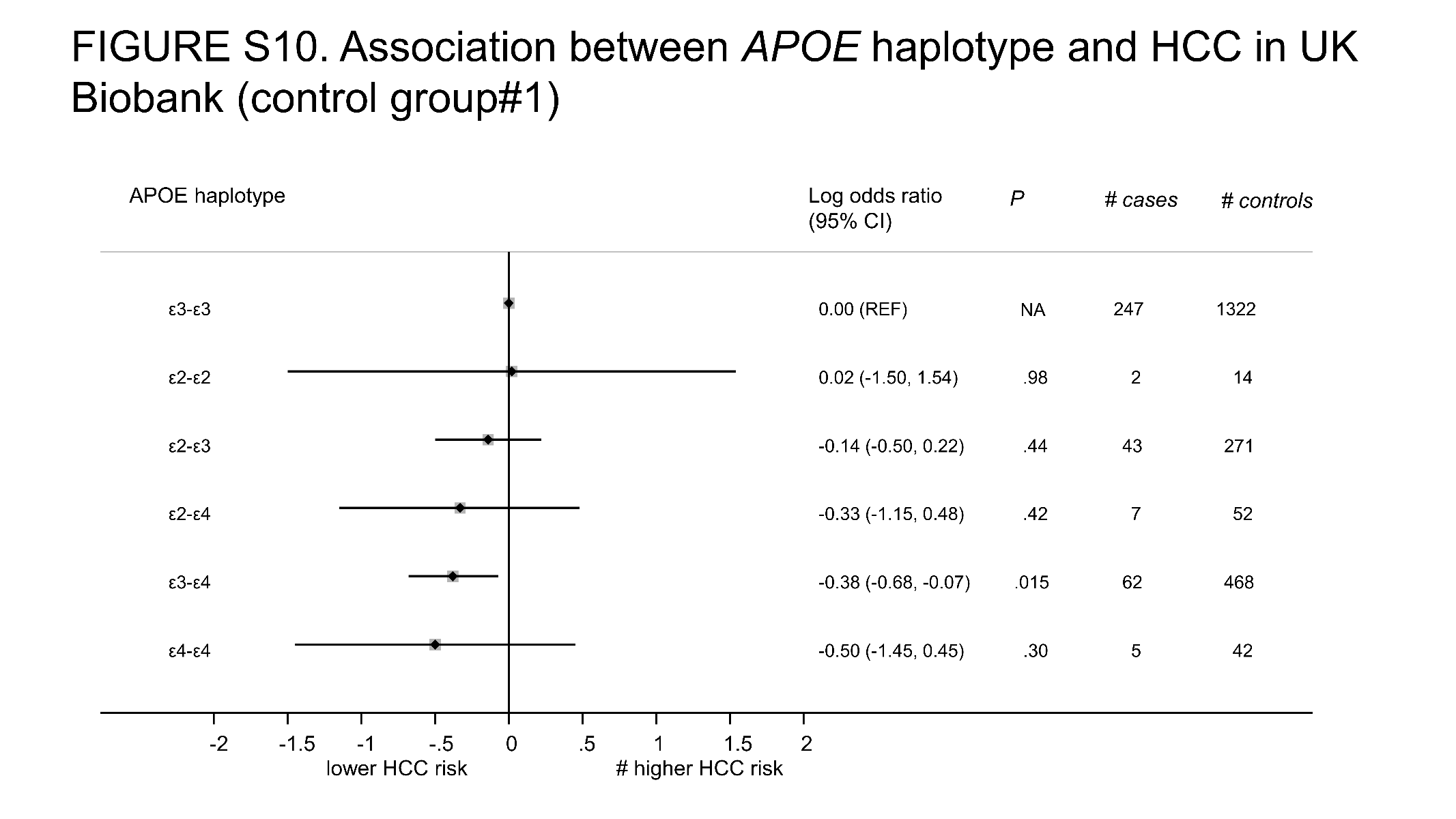


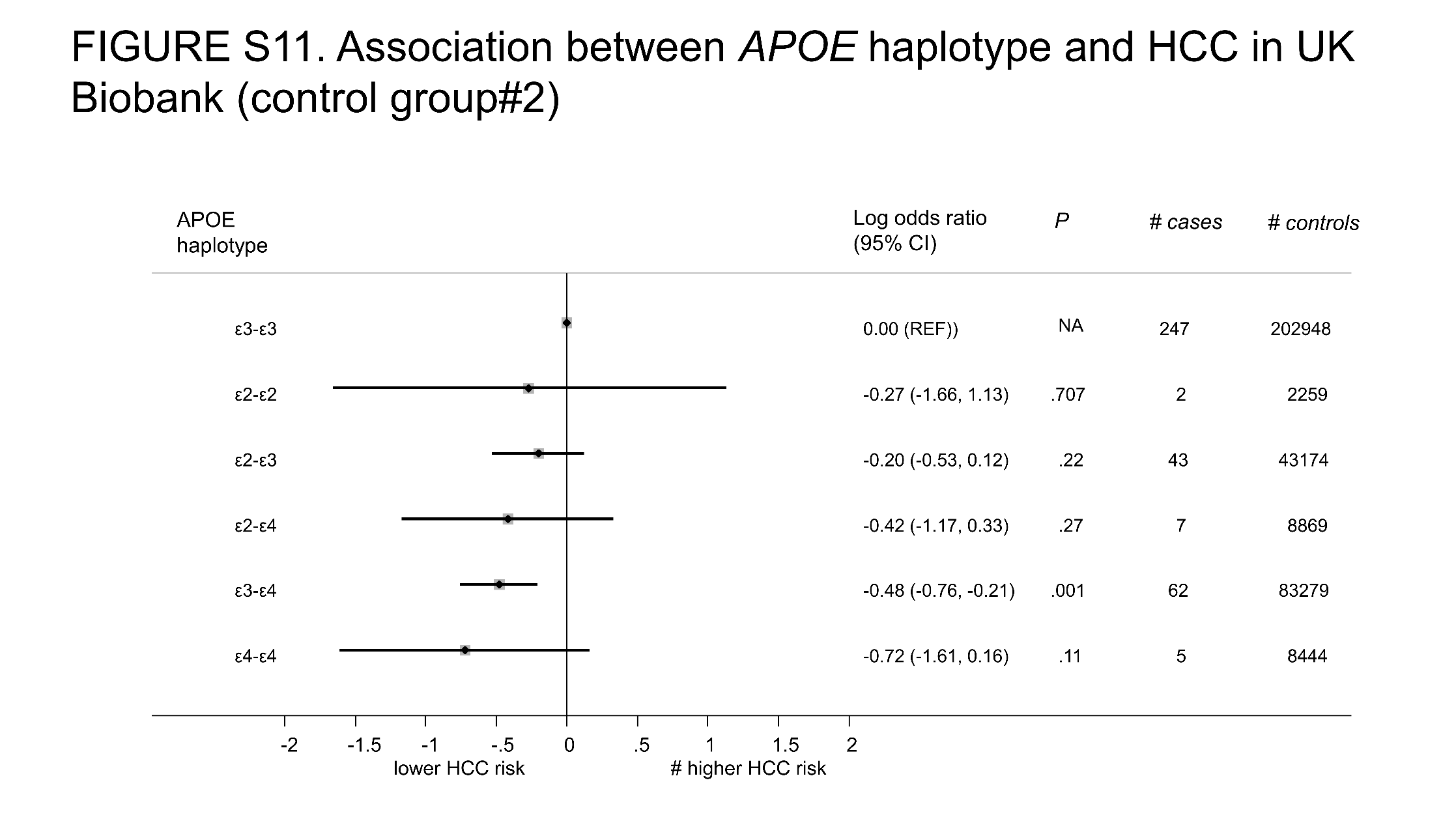


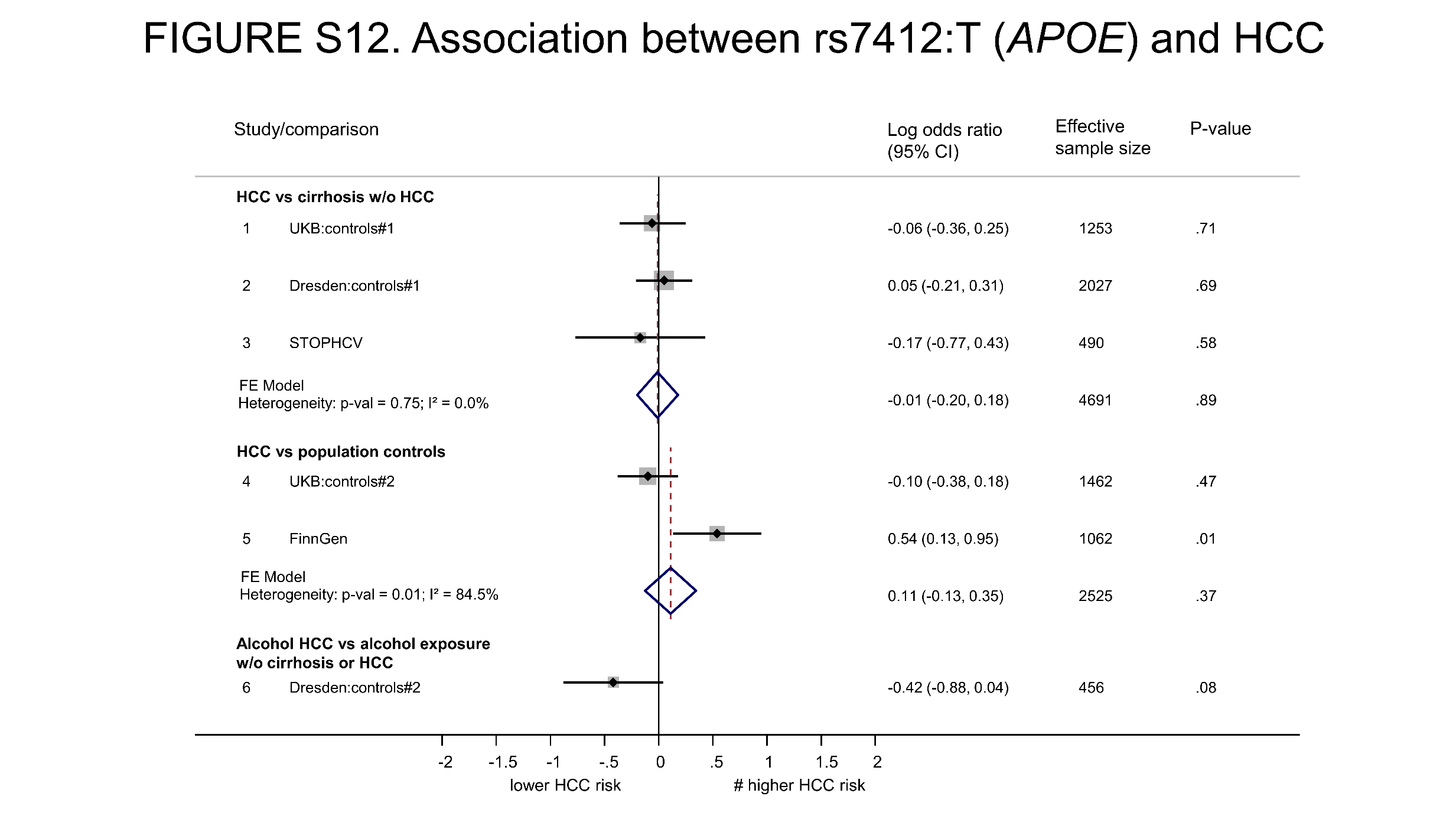

**Appendix A: Genotyping Methods**

**Dresden Cohort:**

Genotyping was performed using the Infinium™ Global Screening Array (GSA)-24 v2.0 BeadChip conducted by the Institute of Clinical Molecular Biology’s DNA Laboratory and Genotyping Core Facilities (IKMB, Kiel, Germany, https://www.ikmb.uni-kiel.de/). Individuals with genotyping success <98%, outlying autosomal heterozygosity (more than 3 s.d. from the mean) or a kinship coefficient (p̂) <0.185 and those failing gender checks were excluded from analysis. We used Plink 2.0 to calculate the first 10 principal components of genetic variation in the genotype data to model subtle ancestry differences between cases and controls along continuous axes of variation. Variants rs58542926 (*TM6SF2*), rs429358 and rs7412 (*APOE*) and rs738409 (*PNPLA3*) were available on the GSA bead chip. Imputation: Genotype data for [rs2642438](http://www.ncbi.nlm.nih.gov/projects/SNP/snp_ref.cgi?rs=2642438) (*MARC1*), rs13130041 (*HSD17B13*), [rs2792751](http://www.ncbi.nlm.nih.gov/projects/SNP/snp_ref.cgi?rs=2792751) (*GPAM*) and [rs187429064](http://www.ncbi.nlm.nih.gov/projects/SNP/snp_ref.cgi?rs=187429064) (*TM6SF2*) were imputed from the Haplotype Reference Consortium reference panel be using the free genotype imputation service of the Michigan Imputation Server (https://imputationserver.sph.umich.edu/index.html#!). All genetic markers were tested for Hardy-Weinberg equilibrium (P>0.05).

**STOPHCV cohort:**

Genotyping was performed using the Affymetrix UK Biobank chip. Individuals with <99% genotype success were excluded. Related individuals were excluded by the identity-by-descent kinship coefficient (p̂) <0.185, implemented in Plink 1.9. Variants rs738409 (*PNPLA3*); rs58542926 (*TM6SF2*); rs72613567(*HSD17B13*); rs429348, rs7412 (*APOE*); rs2642438 (*MARC1*); rs2792751 (*GPAM*) were included on the Affymetrix chip and hence were genotyped directly. Genotype data for rs187429064 (*TM6SF2*) variant was imputed from the Haplotype Reference Consortium reference panel using the Sanger imputation server (https://www.sanger.ac.uk/tool/sanger-imputation-service/). Variants with a violation of Hardy Weinberg equilibrium (*P*<1 x 10^-6^) were removed prior to imputation. We used Plink 2.0 to calculate principal components of genetic ancestry.

**UK Biobank**

UK Biobank genotyping methods have been described in detail previously by Bycroft et al.[1]

**FinnGen:**

Genotyping methods for the FinnGen cohort are outlined in detail here: <https://www.finngen.fi/en/researchers/genotyping>

REFERENCE:

[1] Bycroft C, Freeman C, Petkova D, Band G, Elliott LT, Sharp K, et al. The UK Biobank resource with deep phenotyping and genetic data. Nature. 2018;562:203-209.

**Appendix B: Polygenic Risk Scores**

Methods

In supplementary analyses, we determined the association between established risk variants – rs738409 in *PNPLA3*; rs58542926 in *TM6SF2* and rs72613567 in *HSD17B13* – and HCC in our cirrhosis-control datasets. Data for rs72613567 were unavailable in the Dresden cohort and so rs13130041 was used as the nearest proxy (R^2^=0.958 in Europeans). Variants were ranked according to their discriminative ability as a single variant – i.e. their ability to discriminate between HCC cases and cirrhosis controls. Discriminative ability was measured using the Concordance-index statistic, which reflects the proportion of evaluable case-control pairs (i.e. where each pair comprises one case and one control), for which the case carries more copies of the risk allele than the control.^1,2^

By extension, we generated polygenic risk scores (PRS) for each cohort, based on different combinations of risk variants and both with and without adjustment for age and sex. Each PRS was derived from the linear predictor of the best-fitting logistic regression model. The likelihood ratio statistic test was used to assess if one model offered a statistically significant improvement in model fit relative to a second nested model. The Concordance-index was calculated for each PRS to assess predictive utility. The relationship between the PRS value and HCC risk was assessed in two ways. First, in terms of the LOR per one standard deviation increase. Second, we categorised patients into risk quintiles (i.e. five equally sized groups with similar PRS values) and calculated the association between risk quintile and HCC status.

Note that due to variability in patient case-mix, one would not necessarily expect the discriminative ability of GRSs to be comparable across datasets. ^3^ Thus, our focus was on quantifying the extent to which the new variants in *APOE* and *TM6SF2* improve model performance relative to GRSs based on established variants only.

Results

As a single variant, the rs429358 in *APOE* variant was ranked #3 in UKB (C-index:0.529); #3 in Dresden (C-index: 0.528) and #1 in STOPHCV (C-index: 0.529) in terms of discriminative ability. The rs187429064 in*TM6SF2* variant exhibited the lowest discriminative ability in the Dresden and STOPHCV cohorts.

The five-variant PRS (comprising new and established variants) offered a statistically significant improvement in model fit versus the three-variant score (comprising established variants only) (see Figure below). Association between the five-variant PRS and HCC varied according to cohort. It was strongest in the Dresden study (LOR per SD increase: 0.50; 95%CI: 0.40-0.59; P=2.1 x 10^-24^; C-index:0.78) and lowest in STOPHCV (LOR per SD increase: 0.22; 95%CI: 0.04-0.40; *P*=0.018; C-index:0.56) (see Figure below).


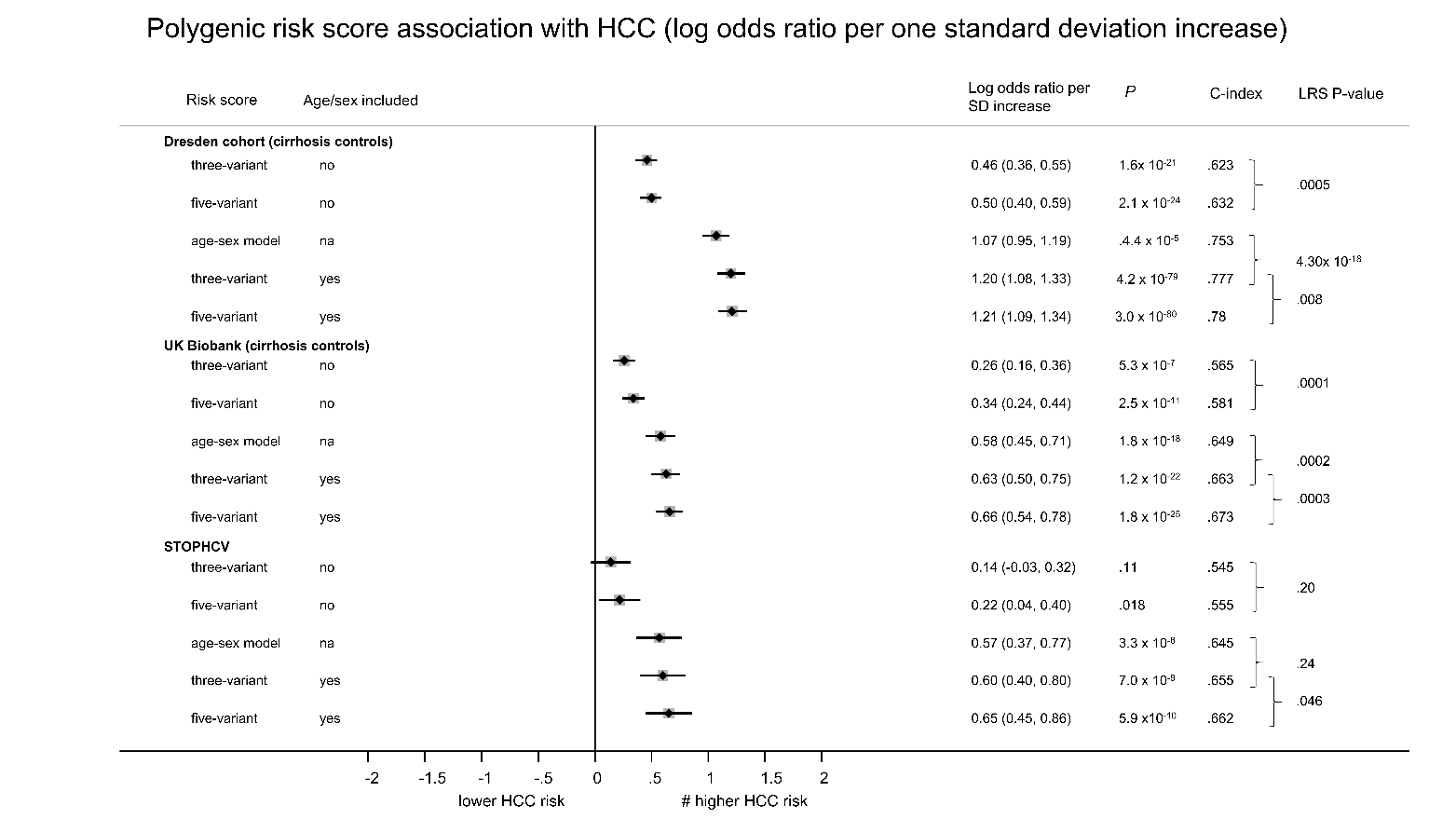


Each five-variant risk score was improved substantially by adding information on age and sex. For example, the C-index increased from 0.632 to 0.780 in the Dresden cohort, from 0.581 to 0.673 in UKB, and from 0.555 to 0.662 in STOPHCV (see Figure above).

The association between risk quintile and HCC status was greatest when comparing quintile 5 (i.e. individuals with a PRS value in the top 20%) against quintile 1 (i.e. the bottom 20%). The OR association for Q5 Versus Q1 was: 2.03 (95%CI: 1.16-3.56; *P*=0.013) in STOPHCV; 3.60 (95%CI: 2.75-4.71; P=3.50 x 10^-20^) in Dresden; and 2.24 (95%CI: 1.66-3.36; *P*=1.7 x 10^-7^) in UKB. These ORs were even higher when age and sex were incorporated into the PRS (See Figure below).


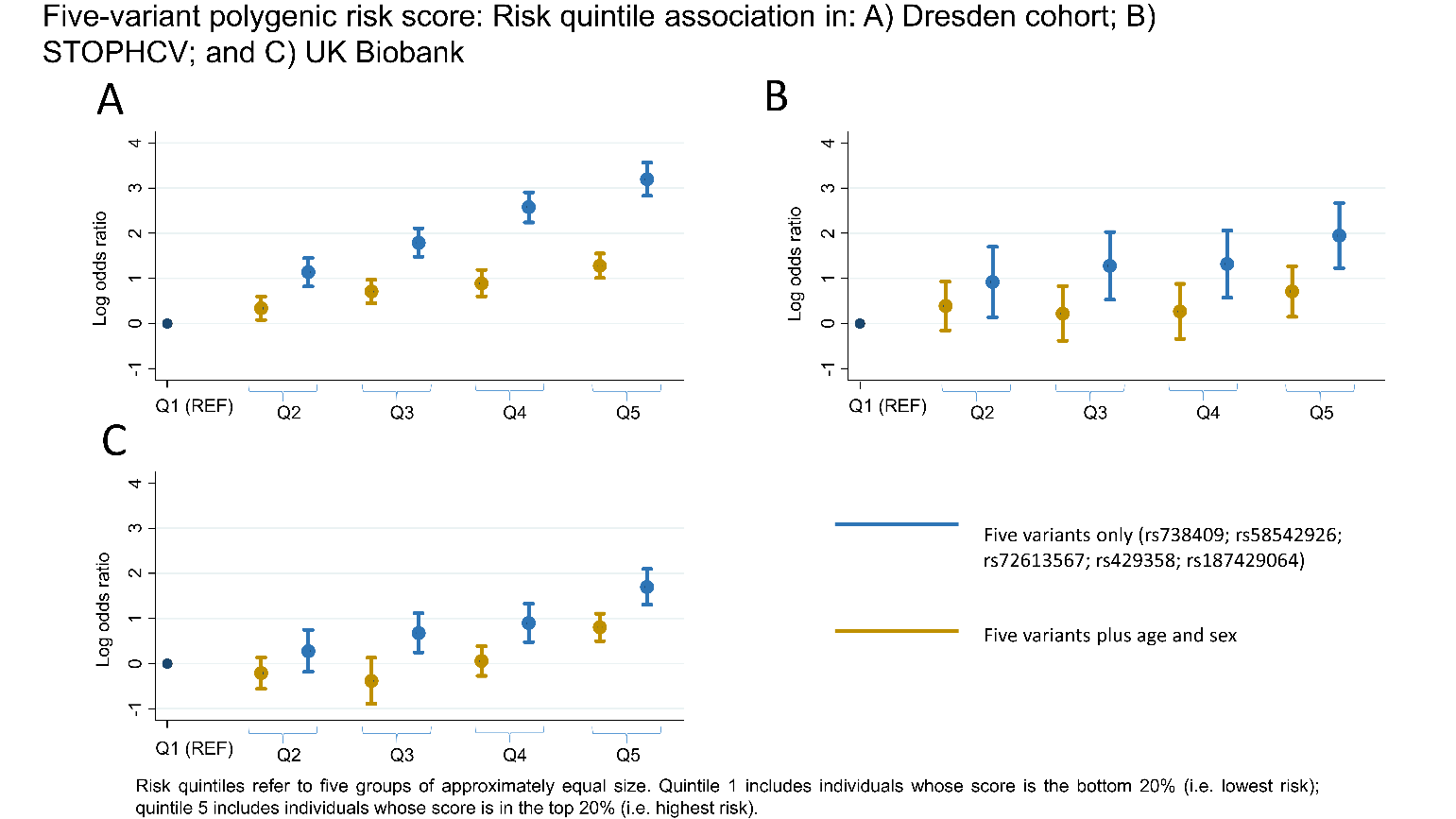


Formulas for all polygenic risks score generated are provided in the table below.

APPENDIX B REFERENCES

[1] Moons KGM, Altman DG, Reitsma JB, Ioannidis JPA, Macaskill P, Steyerberg EW, et al. Transparent reporting of a multivariable prediction model for individual prognosis or diagnosis (TRIPOD): explanation and elaboration. Ann Intern Med. 2015;162:W1-73.

[2] Steyerberg EW. Clinical Prediction Models: a practical approach to development, validation and updating. Springer. 2009.

[3] Damen JAAG, Debray TPA, Pajouheshina R, Reitsma JB, Scholten RJPM, Moons KGM, Hooft L. Empirical evidence of the impact of study characteristics on the performance of prediction models: a meta-epidemiological study. BMJ Open. 2019;1:e026160.
